# Increased blood glycohemoglobin A1c levels lead to overestimation of arterial oxygen saturation by pulse oximetry in patients with type 2 diabetes

**DOI:** 10.1186/1475-2840-11-110

**Published:** 2012-09-17

**Authors:** Li Jin Pu, Ying Shen, Lin Lu, Rui Yan Zhang, Qi Zhang, Wei Feng Shen

**Affiliations:** 1 Department of Cardiology, Shanghai Rui Jin Hospital, Shanghai, 200025, People’s Republic of China; 2 Institute of Cardiovascular Diseases, School of Medicine, Shanghai Jiaotong University, Shanghai, 200025, People’s Republic of China

**Keywords:** Glycohemoglobin A1c, Diabetes mellitus, Arterial blood gas analysis, Pulse oxygen saturation

## Abstract

**Background:**

Non-enzymatic glycation increases hemoglobin-oxygen affinity and reduces oxygen delivery to tissues by altering the structure and function of hemoglobin.

**Objectives:**

We investigated whether an elevated blood concentration of glycosylated hemoglobin (HbA1c) could induce falsely high pulse oximeter oxygen saturation (SpO_2_) in type 2 diabetic patients during mechanical ventilation or oxygen therapy.

**Methods:**

Arterial oxygen saturation (SaO_2_) and partial pressure of oxygen (PO_2_) were determined with simultaneous monitoring of SpO_2_ in 261 type 2 diabetic patients during ventilation or oxygen inhalation.

**Results:**

Blood concentration of HbA1c was >7% in 114 patients and ≤ 7% in 147 patients. Both SaO_2_ (96.2 ± 2.9%, 95% confidence interval [CI] 95.7-96.7% vs. 95.1 ± 2.8%, 95% CI 94.7-95.6%) and SpO_2_ (98.0 ± 2.6%, 95% CI 97.6-98.5% vs. 95.3 ± 2.8%, 95% CI 94.9-95.8%) were significantly higher in patients with HbA1c >7% than in those with HbA1c ≤ 7% (Data are mean ± SD, all p < 0.01), but PO_2_ did not significantly differ between the two groups. Bland-Altman analysis demonstrated a significant bias between SpO_2_ and SaO_2_ (1.83 ±0.55%, 95% CI 1.73% -1.94%) and limits of agreement (0.76% and 2.92%) in patients with HbA1c >7%. The differences between SpO_2_ and SaO_2_ correlated closely with blood HbA1c levels (Pearson’s r = 0.307, p < 0.01).

**Conclusions:**

Elevated blood HbA1c levels lead to an overestimation of SaO_2_ by SpO_2_, suggesting that arterial blood gas analysis may be needed for type 2 diabetic patients with poor glycemic control during the treatment of hypoxemia.

## Background

Glycohemoglobin is produced via a non-enzymatic reaction between the free aldehyde group of glucose or other sugars and the unprotonated form of free amino groups of hemoglobin [1]. Glycosylated hemoglobin A1c (HbAlc) is a stable minor hemoglobin variant separated by charge that is composed primarily but variably of glycohemoglobin. A clinical relationship between blood concentration of HbAlc and status of glycemic control has been elucidated [2], and elevated HbAlc levels represent increased risk of coronary artery disease and poor outcome in diabetic patients [3-5]. Previous studies have shown that glycosylation alters the structure and function of hemoglobin [6,7] and tends to shift the oxygen dissociation curve to the left, leading to an increase in hemoglobin-oxygen affinity and a reduction in oxygen delivery to tissues [6,8,9]. Pulse oximetry is widely used as a noninvasive tool for continuous monitoring of artery oxygen saturation (SaO_2_) [10,11], but pulse oximeter oxygen saturation (SpO_2_) may overestimate arterial blood gases-determined SaO_2_ in acute sickle chest syndrome and severe sepsis [12,13]. So far, its accuracy for titrating fractional inspired oxygen in type 2 diabetic patients with mechanical ventilation or oxygen therapy remains unclear. Given that chronic hyperglycemia accelerates the accumulation of advanced glycation end products (AGE) in the skin collagen [14], which poses specific autofluorescence feature, may emit light by absorbing specific wavelengths light [15], and interfere with the accuracy of pulse oximetry, it is pertinent to examine if elevated blood HbAlc concentrations could result in an overestimation of SaO_2_ by SpO_2_ with finger probes particularly for type 2 diabetic patients with poor glycemic control.

## Methods

### Study population

A total of 261 consecutive type 2 diabetic patients undergoing oxygen therapy and/or mechanical ventilation from October 2010 to May 2012 in Rui Jin Hospital, Shanghai Jiaotong University School of Medicine were included. None had any recorded history of carbon monoxide exposure. The diagnosis of type 2 diabetes was made according to the criteria of American Diabetes Association, including symptoms of diabetes plus casual plasma glucose concentration beyond 200 mg/dl (11.1 mmol/l), or an increased fasting (126 mg/dl [7.0 mmol/l]) or 2-hour postprandial glucose (2-h PG) level (200 mg/dl [11.1 mmol/l] during an oral glucose tolerance test) [16]. Patients with type 1 diabetes were excluded by measurement of C-peptide. For the purpose of this study, we also excluded patients who were current cigarette smokers within 3 months or alcohol-dependent, had renal insufficiency, anemia and fever, or were treated with vaso-constrictive agents. Poor glycemic control was defined as blood HbAlc level >7% [17]. The study was approved by the hospital Institutional Review Board (IRB), and written informed consent was obtained from all participants.

### Biochemical assessments

Peripheral venous blood was collected after an overnight fasting in all patients, and serum levels of glucose, blood urea nitrogen, creatinine, total cholesterol, low-density lipoprotein-cholesterol (LDL-C), high-density lipoprotein cholesterol (HDL-C) and triglycerides were measured with standard laboratory techniques on a Hitachi 912 Analyzer (Roche Diagnostics, Germany). Hemoglobin concentrations were determined with a model T-890 Coulter (Beckman Coulter International, Nyon, Switzerland). In order to exclude individuals with abnormal hemoglobin, electrophoresis was carried out according to the method of Marengo-Rowe [18]. Blood HbA1c concentration was assayed using ion-exchange high performance liquid chromatography with a Bio-Rad Variant Hemoglobin Testing System (Bio-Rad Laboratories, Hercules, CA, USA). Levels of 2, 3-diphosphoglycerate (2,3-DPG) in the red blood cells were assayed within 15 min using enzymatic determination at 340 nm with an ultraviolet test kit from Roche Diagnostics (Roche Diagnostics GmbH, Mannheim, Germany) according to the manufacturer’s instructions.

### Arterial blood gas analysis

Arterial blood gases were determined during simultaneous SpO_2_ monitoring with pulse oximetry. Blood was drawn anaerobically into a preheparinized 1-mL syringe, and mixed well before measurement. After removal of all air bubbles from the syringe, *in vivo* pH, partial pressure of carbon dioxide (PCO_2_), SaO_2_, partial pressure of oxygen (PO_2_) and carboxyhemoglobin were directly measured using a Cobas b 221 blood gas analyzer (Roche Diagnostics, Germany). All measurements were completed within 5 min of blood sampling. Arterial blood gas analysis and SpO_2_ values were one measurement per patient.

### Monitoring of pulse oxygen saturation

SpO_2_ was monitored continuously with a pulse oximetry (Nellcor NPB 40 MAX, Hayward, California), which detects oxygen saturation by measuring transdermal light absorption in the blood flow through a fingertip (DS 100A finger probe). SpO_2_ values were recorded only after a consistent reading, with a strong arterial waveform signal and a pulse reading identical to the patient’s heart rate.

### Statistical analysis

Baseline characteristics are expressed as mean and standard deviation (SD) for continuous variables, and percentages for categorical ones. Chi-square test was used to analyze dichotomous variables. Comparisons of continuous variables between groups were made by the appropriate Student’s t tests. Correlation between difference (SpO_2_ -SaO_2_) and HbA1c was analyzed using Pearson correlation coefficients. Accuracy (SpO_2_ - SaO_2_) of SpO_2_ was examined by the method of Bland and Altman analysis [19]. Bias was determined by the mean difference and 95% confidence intervals (CI) between SpO_2_ and SaO_2_, precision was determined by the standard deviation of the mean difference, and limits of agreement (mean difference ±1.96SD) was defined as a proportional function of distribution for differences between the 2 measurements. Data were analyzed using the Statistical Packages for Social Sciences (SPSS Version 13.0, Chicago, Ill). A 2-tailed p value < 0.05 was considered statistically significant. Only one measurement per patient for arterial blood gas and SpO_2_ was made.

## Results

### Clinical characteristics

Among overall 261 type 2 diabetic patients, 114 patients had a HbAlc >7%, and 147 had a HbA1c ≤ 7%. Patients with HbAlc >7% were older and had higher serum levels of fasting glucose, 2-h postprandial glucose, and triglycerides than those with HbA1c ≤ 7%. The two groups did not differ with respect to occurrence rates of hypertension, chronic obstructive pulmonary disease, and pulmonary edema (Table 1).

**Table 1:** Baseline characteristiecs and biochemical assessments

**Variables**	**HbA1c≤7% (n=147)**	**HbA1c>7% (n=114)**	**P Value**
Male gender (%)	104(70.7)	78(68.4)	0.685
Age (yrs)	67±6	69±7	0.01
Chronic obstructive pulmonary disease (%)	98(66.7)	81(71.1)	0.449
Cardiogenic pulmonary edema (%)	13(8.8)	17(14.9)	0.127
Pneumonia (%)	40(27.2)	46(40.4)	0.025
Hypertension (%)	49(33.3)	40(35.1)	0.243
Systolic blood pressure (mmHg)	131±9	132±9	0.374`
Diastolic blood pressure (mmHg)	75±8	76±8	0.317
Red blood cell (x10^12^/L)	4.75±0.73	4.91±0.77	0.088
Hemoglobin (g/L)	132±12	134±13	0.199
Body temperature (°C)	37.3±0.1	37.4 ± 0.1	<0.001
Total cholesterol (mmo1/L)	4.06±0.91	4.17±0.95	0.343
HDL-cholesterol (mmo1/L)	1.19±0.26	1.17±0.27	0.545
LDL-cholesterol (mmo1/L)	2.43±0.62	2.41±0.68	0.805
Triglycerides (mmo1/L)	1.72±0.75	1.91± 0.78	0.047
Fasting Glucose (mmo1/L)	5.9±1.8	9.4±2.1	<0.001
2-h postprandial glucose (mmo1/L)	9.2±4.1	13.9± 4.5	<0.001
HbA1c (%)	5.6±1.2	9.2±2.1	<0.001
Blood urea nitrogen (mmo1/L)	5.42±2.35	6.03±2.51	0.045
Creatinine (μmo1/L)	87±16	91±18	0.59
Medical treatments			
ACEI or ARB (%)	46(31.3)	37(32.5)	0.841
Calcium channel blocker (%)	14(9.5)	12(10.5)	0.789
Statins* (%)	121(82.3)	97(85.1)	0.549
Metformin (%)	52(35.4)	45(39.5)	0.497
Sulphonylureas (%)	56(38.1)	37(32.5)	0.345
Alpha-glucosidase (%)	54(36.7)	46(40.3)	0.551
PPAR-gamma agonist (%)	23(15.6)	15(13.2)	0.572

Data are mean ± SD or number (%).

Abbreviation: HbA1c, Glycosylated hemoglobin A1c; HDL, High-density lipoprotein.

LDL, low-density lipoprotein; ACEI, andgiotensin-converting enzyme inhibitor.

ARB, angiotensin II receptor blocker.

*Statins: mainly simvastatin, pravastatin and atorvastatin.

### Arterial blood gas profiles, pulse oximetry, and 2, 3-DPG

Both SaO_2_ (96.2 ± 2.9%, 95% confidence interval [CI] 95.7-96.7% vs. 95.1 ± 2.8%, 95% CI 94.7-95.6%) and SpO_2_ (98.0 ± 2.6%, 95% CI 97.6-98.5% vs. 95.3 ± 2.8%, 95% CI 94.9-95.8%) were significantly higher in patients with HbA1c >7% than in those with HbA1c ≤ 7% (Data are mean ± SD, all p < 0.01), but PO_2_ did not significantly differ between the two groups.

Levels of 2, 3-DPG in the red blood cells and PCO_2_ were slightly elevated in patients with HbA1c >7%, but did not reach statistical significance levels (p >0.05). Body temperature, pH, and carboxyhemoglobin were similar in the two groups (Table 2).

**Table 2:** **Arterial blood gas analysis, 2,3-DPG level and SpO**_**2**_**between two groups**

**Variables**	**HbA1c≤7% (n=147)**	**HbA1c>7% (n=114)**	**P Value**
2, 3-DPG(μmo1/gHb)	13.9±1.7	14.2±1.7	0.09
pH	7.37±0.05	7.39±0.05	0.01
PCO_2_ (Kpa)	5.48±0.73	5.53±0.65	0.565
Carboxyhemoglobin (%)	1.08±0.81	1.09±0.78	0.92
PO_2_ (Kpa)	10.0±1.9	10.0±2.2	0.843
SaO_2_ (%)	95.1±2.8	96.2±2.9	0.002
SpO_2_ (%)	95.3±2.8	98.0±2.6	0.001

Data are mean ± SD.

Abbreviation: 2,3-DPG, 2, 3-diphosphosglycerate.

PCO_2_, partial pressure of carbon dioxide.

PO_2_, partial pressure of oxygen; SaO_2_, arterial oxygen saturation.

SpO_2_, pulse oximeter oxygen saturation.

**Figure 1:**
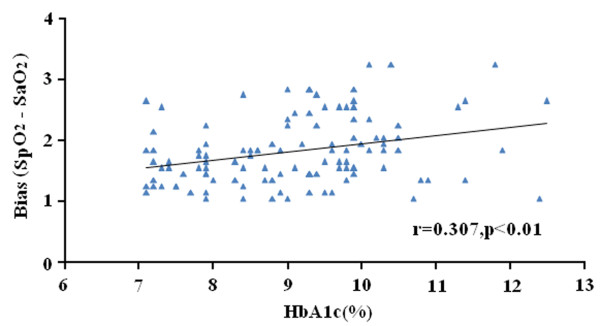
**Correlation of the difference between SpO**_**2**_**and SaO**_**2**_**with blood HbA1c levels in patients with poor glycemic control.**

The difference between SpO_2_ and SaO_2_ correlated closely with blood HbA1c levels (Pearson’s r = 0.307, p < 0.01) (Figure 1).

### Arterial oxyhemoglobin sigmoid curves

**Figure 2:**
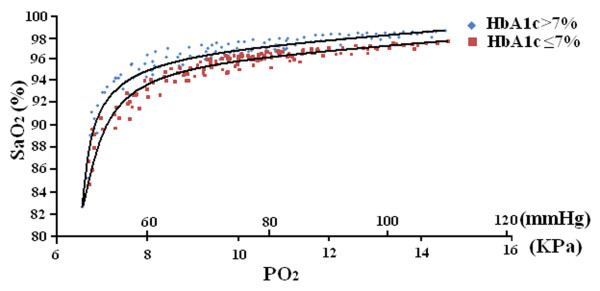
**Arterial oxyhemoglobin sigmoid curves (PO**_**2**_**, partial pressure of oxygen; SaO**_**2,**_**arterial oxygen saturation).**

The sigmoid fitted curve for patients with HbA1c >7% shifted to the left compared with that with HbA1c ≤ 7%. The mean difference of SaO_2_ between diabetic patients with HbA1c >7% and those with HbA1c ≤ 7% was 1.1% (Figure 2).

### Bland-Altman analysis

**Figure 3:**
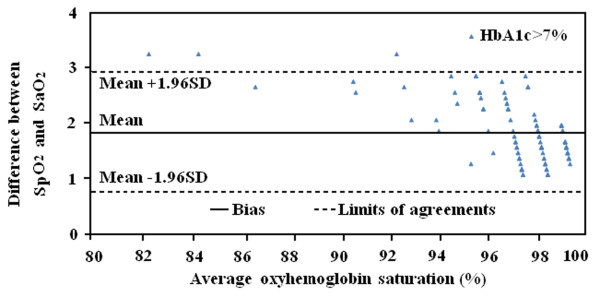
Bland-Altman plots for bias and limits of agreement in patients with poor glycemic control.

The simultaneous readings of SaO_2_ and SpO_2_ were analyzed to determine the bias and limits of agreement. Bland-Altman analysis indicated that the bias (mean difference of SpO_2_ minus SaO_2_) between the two methods was 1.83 ±0.55% (95% CI1.73% -1.94%) and limits of agreement were 0.76% and 2.92% in patients with HbA1c >7% (Figure 3). Overall, there was a significant bias between pulse oximetry and arterial blood gases in patients with HbA1c >7%.

## Discussion

The present study is the first to demonstrate that despite similar levels of red blood cell 2, 3-DPG, PO_2_, PCO_2_, pH and body temperature, type 2 diabetic patients with HbA1c > 7% had higher SaO_2_ (the mean difference was 1.1%) and bias (1.83 ±0.55%) compared with those with HbA1c ≤ 7%, suggesting that elevated blood HbAlc levels led to an overestimation of SaO_2_ by pulse oximetry.

Hypoxemia denotes a condition that is characterized by low oxygen content or percent saturation of hemoglobin with oxygen [13]. Arterial blood gases have been traditionally used to assess the status of oxygenation and to adjust fractional inspired oxygen in patients receiving mechanical ventilation or oxygen therapy [20]. Currently, noninvasive continuous monitoring of SaO_2_ with SpO_2_ has become the standard care for patients with critical conditions [13,21], to decrease the likelihood of hypoxemia [22-25] and to wean mechanical ventilation [10,11,26]. In the present study, when comparisons were performed at identical PO_2_, SaO_2_ was higher in type 2 diabetic patients with HbA1c > 7%, which is likely due to an increased hemoglobin-oxygen affinity. Our results are in line with previous findings that higher blood concentrations of HbA1c significantly reduce oxygen dissociation velocity [27]. Although the exact mechanism remains not fully understood, it may be, at least partly, explained by glycation of multiple ß-chain sites of hemoglobin A molecule, accompanied by increasing -chain glycation at high glycohemoglobin concentrations [28,29].

The major finding of this study is that in type 2 diabetic patients with poor glycemic control, pulse oximetry overestimated arterial blood gases-determined SaO_2_ by a mean of 2.7% when compared with those with HbA1c ≤ 7%. Previous studies showed that older women have higher HbA1c than men, even after controlling for body mass index [30], and accumulation of AGE in human skin collagen is age-dependent [31]. However, both gender distribution and age did not significantly differ between the two groups in the present study, suggesting that the difference between SpO_2_ and SaO_2_ may be mainly related to HbA1c levels as higher HbA1c levels were associated with great differences (Pearson’s r = 0.307, p < 0.01).

Our findings may be of important clinical relevance. First, falsely high SpO_2_ could cause under-diagnosis of hypoxemia in type 2 diabetic patients. Second, because a greater SpO_2_ was required to achieve the same arterial blood gases-determined PO_2_ for diabetic patients with HbA1c >7% compared with those with HbA1c ≤ 7%, care should be taken in adjusting oxygen supply during mechanical ventilation or oxygen therapy. The reason for a higher SpO_2_ than SaO_2_ may be partly explained by an extensive accumulation of AGE in the skin collagen in patients with poor glycemic control [14], interfering with transdermal absorption of the specific wavelength light by hemoglobin with finger probes [12,32]. These observations support a notion that the causes of high bias does include skin effect [33,34], and when SaO_2_ needs to be determined with a high degree of accuracy, arterial blood gases are recommended in type 2 diabetic patients with poor glycemic control.

### Limitations

Due to relatively small sample size, potential for selection bias may raise some concerns on the statistical precision of the estimates. A large-scale study is warranted to confirm our findings. The other major limitation is that most of the data are at high SaO_2_ because of a specially selected study population as all patients were receiving mechanical ventilation and/or oxygen therapy. The oxyhemoglobin dissociation curves could actually be fitted with non-linear regression, and a partial pressure of oxygen in blood associated to a hemoglobin oxygen saturation of 50% (P50) could also be calculated [35]. Most oximetry testing intentionally gathers data below 90% by performing desaturation experiments in volunteers. Gather multiple data points on volunteers increase the data set substantially, and allow one to test over a wide range of SaO_2_, and control for other effects. By creating a gas pocket with CO_2_ and O_2_/N_2_ mixtures, one can create a much more detailed oxyhemoglobin dissociation curve. The US FDA requires testing balanced by gender and ethnicity. Repeated measures statistics would then be necessary, and P50 is not determined quite as precisely unless the sample is near a SaO_2_ of 50%. Certainly, it remains unclear whether the issue with diabetic patients would be safe given the possibility of cardiovascular disease, although younger subjects could be reasonable.

## Conclusions

Elevated blood HbA1c concentrations lead to an overestimation of SaO_2_ by SpO_2_, suggesting that arterial blood gas analysis may be needed for type 2 diabetic patients with poor glycemic control during the treatment of hypoxemia.

## Abbreviations

AGE: Advanced glycation end products; 2, 3-DPG: 2, 3-diphosphoglycerate; CI: Confidence interval; HbAlc: Glycosylated hemoglobin A1c; HDL: High-density lipoprotein; LDL: Lower-density lipoprotein; PCO_2_: Partial pressure of carbon dioxide; PO_2_: Partial pressure of oxygen; SaO_2_: Arterial oxygen saturation; SD: Standard deviation; SpO_2_: Pulse oximeter oxygen saturation.

## Competing interests

The authors declare that they have no competing interests.

## Authors' contributions

LJP designed the study and drafted the manuscript; YS collected the samples and performed the experiments; LL participated in the whole process of experiments; RYZ and QZ contributed to data analysis; WFS was responsible for the whole research project and prepared the manuscript. All authors have read and approved the final manuscript.
